# A mobile phone intervention to reduce binge drinking among disadvantaged men: study protocol for a randomised controlled cost-effectiveness trial

**DOI:** 10.1186/1745-6215-15-494

**Published:** 2014-12-19

**Authors:** Iain K Crombie, Linda Irvine, Brian Williams, Falko F Sniehotta, Dennis Petrie, Josie MM Evans, Carol Emslie, Claire Jones, Ian W Ricketts, Gerry Humphris, John Norrie, Peter Rice, Peter W Slane

**Affiliations:** Division of Population Health Sciences, School of Medicine, University of Dundee, Dundee, DD2 4BF UK; Nursing, Midwifery and Allied Health Professions Research Unit, University of Stirling, Stirling, FK9 4LA UK; Institute of Health & Society, University of Newcastle, Newcastle, NE2 4AX UK; Centre for Health Policy, Programs and Economics, University of Melbourne, Melbourne, Australia; School of Health Sciences, University of Stirling, Stirling, FK9 4LA UK; School of Health and Life Sciences, Glasgow Caledonian University, Glasgow, G4 0BA UK; Health Informatics Centre, University of Dundee, Dundee, DD1 9SY UK; School of Computing, University of Dundee, Dundee, DD1 4HN UK; School of Medicine, University of St Andrews, St Andrews, KY16 9TF UK; Centre for Health Care Randomised Trials, University of Aberdeen, Aberdeen, AB25 2ZD UK; Division of Neuroscience, University of Dundee, Dundee, DD1 9SY UK; Arthurstone Medical Centre, Dundee, DD4 6QY UK

**Keywords:** Binge drinking, Disadvantaged men, Mobile phone messages, Randomised controlled trial

## Abstract

**Background:**

Socially disadvantaged men are at a substantially higher risk of developing alcohol-related problems. The frequency of heavy drinking in a single session is high among disadvantaged men. Brief alcohol interventions were developed for, and are usually delivered in, healthcare settings. The group who binge drink most frequently, young to middle-aged disadvantaged men, have less contact with health services and there is a need for an alternative method of intervention delivery. Text messaging has been used successfully to modify other adverse health behaviours. This study will test whether text messages can reduce the frequency of binge drinking by disadvantaged men.

**Methods/design:**

Disadvantaged men aged 25 to 44 years who drank >8 units of alcohol at least twice in the preceding month will be recruited from the community. Two recruitment strategies will be used: contacting men listed in primary care registers, and a community outreach method (time-space sampling). The intended sample of 798 men will be randomised to intervention or control, stratifying by recruitment method. The intervention group will receive a series of text messages designed to reduce the frequency of binge drinking through the formation of specific action plans. The control group will receive behaviourally neutral text messages intended to promote retention in the study. The primary outcome measure is the proportion of men consuming >8 units on at least three occasions in the previous 30 days. Secondary outcomes include total alcohol consumption and the frequency of consuming more than 16 units of alcohol in one session in the previous month. Process measures, developed during a previous feasibility study, will monitor engagement with the key behaviour change components of the intervention. The study will incorporate an economic evaluation comparing the costs of recruitment and intervention delivery with the benefits of reduced alcohol-related harm.

**Discussion:**

This study will assess the effectiveness of a brief intervention, delivered by text messages, aimed at reducing the frequency of binge drinking in disadvantaged men. The process measures will identify components of the intervention which contribute to effectiveness. The study will also determine whether any benefit of the intervention is justified by the costs of intervening.

**Trial registration:**

ISRCTN07695192. Date assigned: 14 August 2013.

## Background

Alcohol-related morbidity and mortality represent a major public health challenge. Estimates of the cost of alcohol to society in England range from £20 to £55 billion per year [1] and is thought to be more than £3.5 billion per year in Scotland [2]. These costs occur through lost productivity, increased healthcare, offending behaviour and other public sector costs and through social disruption. Alcohol-related harms are not evenly distributed in the population. Levels of binge drinking, heavy drinking in a single session, are highest among young and disadvantaged men [3]. People who are socially disadvantaged are also at a substantially higher risk of developing alcohol-related diseases [4, 5].

Brief interventions, based on psychological theories of behaviour change, have been developed to tackle alcohol-related problems. There is extensive evidence that they are effective [6–9]. However, these interventions were developed for and are usually delivered in healthcare settings. The group who binge drink most frequently, young to middle-aged disadvantaged men [3], are seldom in contact with health services. They are therefore much less likely to be reached by current initiatives to tackle excessive drinking. A further challenge is that the uptake of public health interventions among socially disadvantaged men is low [10]. There is a need for an intervention which effectively reduces binge drinking in this hard-to-reach population.

Text messaging provides a method for delivering brief alcohol interventions which can reach large numbers of individuals at low cost. Text messaging has been used successfully to modify other adverse health behaviours [11, 12] and to increase healthcare uptake [13, 14]. This approach is particularly well suited to young to middle-aged men because their ownership of mobile phones is high. Recent systematic reviews have shown that text messages can successfully change behaviour [15–17]. However, no studies have tackled binge drinking and none have been targeted at disadvantaged men.

We recently completed an National Institute for Health Research funded feasibility study which demonstrated that all stages of a trial of a brief intervention delivered by mobile phone could be completed successfully [18]. It identified a high frequency of hazardous drinking among disadvantaged men and a pressing need for effective interventions to tackle their binge drinking. The success of the feasibility study led to funding for a full trial to assess the effectiveness and cost effectiveness of the intervention. This protocol describes the full trial.

## Methods/design

### Overview

**Figure 1:**
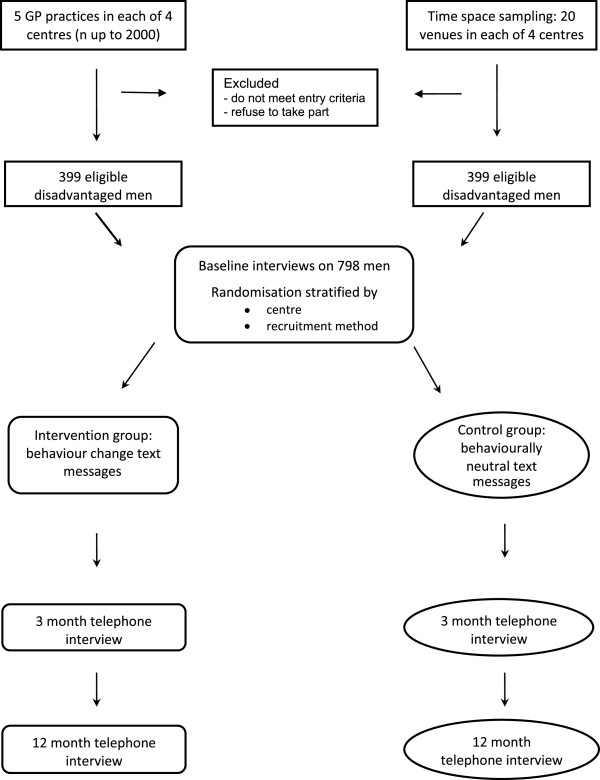
**Consort flow diagram.**

This study will determine whether a brief intervention delivered by mobile phone can reduce the frequency of binge drinking among disadvantaged men. It is a four centre, parallel group, randomised, double-blind, controlled trial. The four centres cover major regions of Scotland: Tayside, Greater Glasgow, Forth Valley and Fife. Participants will be recruited through two strategies: through primary care registers and by time-space sampling. Randomisation will be stratified by recruitment method. The study will incorporate an economic evaluation to assess the cost effectiveness of the intervention. A flowchart of the recruitment and follow-up is shown in Figure 1. Ethical approval for all aspects of the study was obtained from the East of Scotland Ethics Service (reference number 13/ES/0058).

### Recruitment strategies

#### Strategy 1

Recruitment through primary care will be supported by the Scottish Primary Care Research Network. Potential participants will be identified from 20 general practitioner (GP) practice lists (five in each of the four centres). These lists contain data on age, gender and postcode. Postcode will be used to derive the Index of Multiple Deprivation score [19]. Potential participants will receive a letter from their GP inviting them to take part. Those who do not opt out will be contacted by telephone. Up to six attempts at contact by phone will be made.

#### Strategy 2

An outreach strategy, time-space sampling, will be employed [20, 21]. Time-space sampling recruits from venues which the target group are known to frequent and involves sampling at selected times of day and days of the week. The approach requires initial fieldwork to identify appropriate venues and suitable times for recruitment. At each of the four centres at least 20 venues will be identified, such as charities that support the long-term unemployed, supermarkets, housing associations, and main shopping streets in disadvantaged areas. Venues will be visited at different times to prevent friendship groups from being included in the study. Potential participants will be approached by research assistants and invited to take part in the study. The approach will be informal, friendly and sensitive to the men’s willingness to discuss participation.

Systematic reviews show that repeated attempts at contact and monetary incentives increase recruitment to research studies [22, 23]. Men who agree to take part will be offered an initial £10 gift voucher to offset any charges incurred by receiving and responding to text messages. They will also be sent a £5 gift voucher every 3 weeks for the duration of the intervention. A £10 voucher will be sent for completing the outcome assessment.

### Inclusion/exclusion criteria

Men aged 25 to 44 years will be recruited from areas of high deprivation. Men will be included in the study if they have had two or more episodes of binge drinking (>8 units in a single session) in the preceding month. Exclusion criteria are men who are currently attending care at an Alcohol Problem Service, and men who will not be contactable by mobile phone for any part of the intervention period.

### Informed consent

Verbal consent will be sought after potential participants confirm that they have understood the contents of the information leaflet. Formal consent will be obtained by text message: potential participants will be asked to reply to a text if they wish to take part. Thus, consent will be the positive action of responding to a text message.

### Randomisation

The randomisation will be carried out using the secure remote web-based system provided by the Tayside Clinical Trials Unit. Randomisation will be stratified by participating centre and the recruitment method and restricted using block sizes of randomly varying lengths.

### Allocation concealment

The randomisation and the delivery of the active and control interventions are by a remote secure system. The researchers who collect the baseline and outcome data will have no access to this system and will be blind to treatment group.

### Baseline assessment

The frequency of binge drinking will be measured using questions taken from the US Behavioural Risk Factor Surveillance System [24]. These questions also enable total consumption of alcohol to be measured. Individual level socio-demographic status will be measured using marital status, employment status, education and housing tenure. By design, few questions on alcohol will be asked at the baseline interview to prevent such questions influencing drinking behaviour in the control group. This possibility was raised by the feasibility study and is supported by recent systematic reviews [25, 26] which show that baseline questions can lead participants to re-evaluate drinking behaviour.

### The intervention

The intervention will be delivered as a series of mobile phone text messages sent over a 12-week period. It will be informed by the Health Action Process Approach (HAPA) [27], a comprehensive model which allows integration of a range of evidence-based behaviour techniques. As with other behaviour change theories, the HAPA addresses pre-intentional motivational processes that lead to behavioural intentions. However, this model suggests that the adoption, initiation, and maintenance of new health behaviour occur as a process that involves a motivational phase and a volitional phase. The volitional phase includes planning, action, and maintenance. Perceived self-efficacy has a crucial role in achieving success in both phases. This approach addresses the intention-behaviour gap, identified as a weakness in some behaviour change models. It emphasises the post-intentional volition that leads to behaviour change. Volition relates closely to a range of effective behaviour change techniques, making the HAPA particularly suitable as an intervention model.

The content of the intervention text messages will also be derived from research findings on: alcohol brief interventions [6–9]; text message interventions [15–17]; Motivational Interviewing [28], Communication Theory [29] and taxonomies of behaviour change techniques [30, 31] including one specific to alcohol brief interventions [32]. The messages will be constructed to take advantage of the conventional pattern of heavy weekend drinking. They will tap into three windows of opportunity: before weekend drinking, after a heavy drinking episode and midweek sobriety. Messages will be tailored to the target group by embedding them in the language and the drinking culture of disadvantaged young men. A variety of techniques will be employed to increase message effectiveness: use of gain-framed texts; pairing of messages; and inclusion of questions to promote interactivity.

The causal model for behaviour change is: generate interest in the study; increase awareness of consumption levels which are defined as harmful; increase awareness of susceptibility to alcohol-related harm; increase motivation to reduce the frequency of binge drinking; alter alcohol expectancies; gain commitment to change; develop goals, action plans and coping plans; increase refusal skills; implement strategies to prevent relapse; reduce frequency of binge drinking. The behaviour change strategy follows four stages with several texts addressing each stage.

#### Stage 1

Stage 1 consists of introductory texts: welcome to the study, establish empathy, raise awareness.

The early texts will foster interest in the study and, following the principles of Motivational Interviewing [28], will empathise with the difficulty of changing drinking behaviour. Humour will be used frequently. Texts will explain the definition of binge drinking and increase the salience of the short-term harms of binge drinking. Other texts will build on this by initiating engagement with the short-term personal and social benefits of reduced drinking.

#### Stage 2

Stage 2 consists of pre-intentional texts: create intention to change.

Following Motivational Interviewing [28] the texts will explore the discrepancy between an individual’s drinking habits (becoming drunk) and the intended aims of drinking (having fun and socialising). Texts will seek to modify alcohol expectancies [33] by promoting engagement with the benefits of moderated drinking and will increase awareness of significant others’ views of reduced drinking. They will also address self-efficacy for refusing drinks and avoiding heavy drinking situations.

#### Stage 3

Stage 3 consists of intentional texts: transform intention into action.

Texts will encourage commitment to goal setting and the formation of specific action plans. They will also encourage identification of barriers to change and the development of coping strategies [27]. This will be aided by presenting common risk situations and possible coping strategies, an approach which has been found to be effective in reducing binge drinking [34].

#### Stage 4

Stage 4 consists of actional texts: sustaining behaviour change.

In this phase texts will increase self-efficacy for maintenance of reduced drinking and support habit formation of less frequent binge drinking. Participants will be encouraged to develop strategies for relapse prevention [27]. The texts will also address recovery self-efficacy. Texts will also prompt rehearsal of responses to risky situations and increase men’s confidence and skills in maintaining behaviour change and recovering from any setbacks in the longer term. The purpose of these texts is to sustain behaviour change by reinforcing self-efficacy for maintenance of long-term behaviour change and augmenting recovery self-efficacy.

### Comparator intervention

The comparator group also will receive text messages for a 12-week period. The texts will be behaviourally neutral, by avoiding the behaviour change strategies employed in the active intervention. They will be designed to maintain interest in the study to ensure that the control group complete the outcome assessments. Several text messages from the control group of the feasibility study will be used. The messages will consist of jokes and interesting or unusual facts about health unrelated to alcohol. They will cover topics such as oral health, the common cold, sexual health, mental health, hearing and foot hygiene.

Text message responses will be monitored daily to identify participants who request to withdraw from the study, who appear distressed or are drinking excessively.

### Outcome measures

The primary outcome will be assessed at 12 months after the intervention is delivered. It is the proportion of men consuming >8 units on at least three occasions in the previous 30 days. This outcome is particularly suitable for disadvantaged men: the feasibility study showed that most of the participants had regular episodes of binge drinking with periods of complete abstinence in between. This makes reduction in the frequency of binge drinking a key target for reducing alcohol-related harm. This measure of consumption is used by national surveys of alcohol consumption [35]. The questions on alcohol consumption were taken from the US Behavioural Risk Factor Surveillance System [24]. To minimise recall bias, gentle prompting will be used to encourage participants to review their drinking over the previous 30 days to identify all drinking occasions and amounts consumed. Outcome assessments use the mobile number to which the texts were sent. If that is unsuccessful a landline number or e-mail, if available, is used.

Five secondary outcomes will be measured. To assess the immediate impact of the intervention one secondary outcome will assess binge drinking at 3 months after the intervention is delivered. The frequency of heavy binge drinking (>16 units in a session) will be recorded at 3 and 12 months from the end of the intervention. This level of drinking was found to be common among disadvantaged men in the feasibility study. The Fast Alcohol Screening Test [36] will be used to determine the frequency of hazardous drinking; it is short and suitable for telephone use. Total consumption of alcohol will be measured to ensure comparability with other brief intervention trials.

#### Process assessment during intervention delivery

The successful methods of process assessment developed for the feasibility study will be used [37]. Fidelity of delivery of the text messages will be monitored electronically. Engagement with the study will be measured by the frequency of responses to questions included in the text messages. Content analysis of the responses given will identify the nature of engagement with components of the behaviour change intervention. The feasibility study showed high levels of engagement among participants in the intervention group, with subjective norms, control beliefs, perceived behavioural control and behavioural intentions. It is not possible to measure these constructs in the control group because their text messages are designed to be behaviourally neutral. Additional text questions will be added for the intervention group to monitor identification of barriers to change, the development of coping strategies, self-efficacy for maintenance of reduced drinking and the development of strategies for relapse prevention.

### Assessment and follow-up

Outcomes will be measured by telephone interview at 3 and 12 months after the delivery of the intervention. Interviewers will be blind to intervention status. The feasibility study demonstrated that a structured questionnaire administered by telephone interview could be used to measure these outcomes.

Several strategies will be employed to maximise completion of the outcome assessments. The final text messages to intervention and control groups will build on the engagement of participants in the study and ask them to keep in touch. Participants will be sent a letter prior to each follow-up assessment to remind them about the follow-up assessment and the gift voucher they will be given for completing the assessment. (Participants’ current addresses are recorded at the start of the study and used to send the participants their gift vouchers.)

### Sample size

The primary endpoint is the proportion of men binge drinking (consuming >8 units in one session) on at least three occasions in the previous 30 days. The sample size calculation focuses on a reduction in the frequency of binge drinking. The feasibility study found that frequent binge drinking was very common in the target group. This type of drinking is likely to be the major cause of harm in disadvantaged men.

The sample size calculation is based on the difference in the proportion of frequent binge drinking between intervention and control groups at the 12-month follow-up assessment. It uses the finding from the baseline interviews from the feasibility study that 57% of men consumed >8 units on at least three occasions in the previous 30 days. The proposed effect size is that the intervention will reduce the frequency of binge drinking from 57% to 46%, a net reduction of 11%. A recent systematic review of conventional brief interventions [8] found an 11% difference in frequency of binge drinking between intervention and control. To detect a reduction in the frequency of binge drinking in this way from 57% to 46% (at the 5% significance level with a power of 80%) would require a sample size of 319 per group or 638 in total. The required sample size was then increased by 20% to allow for losses to follow-up, making the total sample size 798. We expect that the loss to follow-up will be less than this, as the loss in our 3-month feasibility study was only 4%. However, as most alcohol brief intervention trials have a loss to follow-up of over 20% [8], it is prudent to make suitable allowance.

### Statistical analysis

The analysis will be by intention to treat. The primary outcome is dichotomous and effect sizes will be presented as proportions of intervention and control groups who have reduced their frequency of binge drinking. Odds ratios and 95% confidence intervals will be calculated. The secondary outcomes will be assessed in a similar way. Further analyses will use logistic regression to explore which of the cognitive antecedents of behaviour change predicts change in the primary outcome. These will include the process measures plus intention to reduce consumption (measured by the Readiness Ruler [38]) and self-efficacy for reducing consumption (using items from the Drinking Refusal Self-efficacy Questionnaire [39]). Goal setting and action planning will be based on the scales developed by Renner and colleagues [40]. The analysis will also explore whether the recruitment method (through primary care or time space sampling) influences treatment effect.

### Economic evaluation

The economic evaluation will take a societal perspective and will model the potential cost effectiveness of the intervention assuming a UK-wide implementation. Resources relating to both recruitment strategies will be collected in addition to the resources required to implement the intervention. These will then be used to predict the costs relating to national rollout where resources will be costed according to their opportunity cost. Health status will be measured by the Euroquol-5D (a health questionnaire with five dimensions) [41], a validated quality of life questionnaire designed to be simple to administer. Assaults, disorderly behaviour, contacts with police/criminal justice, plus accident and emergency and other healthcare usage will be measured by the widely used short Service Use Questionnaire (courtesy of S Parrot at the Department of Health Sciences, University of York, York, UK). The longer term impact on quality-adjusted life years (QALYs) and other harms will be modelled using quantified relationships between consumption (mean weekly consumption and peak daily consumption) and alcohol-attributable harms [42]. The cost effectiveness will then be presented as the cost per person reducing their binge drinking and as the cost per QALY saved. Subgroup differences in cost effectiveness will be explored for the two recruitment strategies. A sensitivity analysis will be conducted to assess the robustness of the conclusions reached to changes in the key assumptions.

## Discussion

This study is the first randomised trial to test the effectiveness of text messages to reduce the frequency of binge drinking by community-recruited disadvantaged men aged 25 to 44 years. It also includes a formal economic evaluation which will provide an estimate of the cost effectiveness of the intervention. This information which will be crucial to the decision by policy makers on whether to adopt this method of tackling alcohol harms. Another ongoing trial also uses texts to reduce binge drinking but it involves younger adults (18 to 25 years) who are attending emergency departments [43]. The results of the two studies will shed light on the extent to which text message interventions delivered to different socio-demographic groups have similar effects.

The intervention has several strengths. It has a very strong empirical and theoretical basis. The intervention provides a detailed causal pathway to behaviour change. The behaviour change strategy aims to increase motivation to change and to maintain longer term behaviour change. It is the first study of its type to address maintenance of changed behaviour. We will be able to investigate this because outcomes of the intervention will be assessed at 3 and 12 months. This will allow us to detect short- and longer term changes in drinking.

A key feature of the intervention is the use of interactive texts in the form of questions to which participants are encouraged to reply. This will reinforce the idea of two-way communication and is intended to promote engagement with key steps in the behaviour change pathway. Our previous research has shown that most men reply to these texts and that the responses provide a non-invasive real-time method to monitor engagement with these key processes [37]. Thus, the analysis will explore whether the extent of engagement predicts the amount of behaviour change. The intervention also uses other techniques to increase engagement, such as tailoring to the target group, timing texts to drinking patterns, gain framing of texts and pairing of text messages.

In this study, the control group will receive a sequence of text messages that are designed to be behaviourally neutral. Trials on alcohol brief interventions vary greatly in the amount of attention given to the control group, with some getting very little attention [8]. In this study the control group will receive a similar number of text messages as the intervention group. The aim is to control for the non-specific effects such as the amount of attention given. Attention could generate a feeling of well-being which could influence the target behaviour, possibly reducing the frequency of binge drinking. In a no-attention control the expectations of participants for some involvement in the study would not be met, leading to a sense of disappointment that could affect, possibly increasing the frequency of binge drinking. In addition, a no-attention control could adversely affect retention in the study. It is not always the case that an attention control should be used [44]. If the non-specific factors are considered an important component of the intervention, then it would be inappropriate to control for them. However, for this study, the hypothesis to be tested was whether the behaviour change strategy in the texts was effective, making an attention control appropriate.

In summary this study aims to address a specific type of hazardous drinking, frequent binge drinking, in a disadvantaged group. The intervention has the potential to be delivered to a large number of community-dwelling individuals at a low cost. If effective it would provide a welcome addition to the conventional alcohol brief intervention delivered in healthcare.

## Trial status

Recruitment started in March 2014 and is ongoing.

## Abbreviations

GP: general practitioner
HAPA: Health Action Process Approach
QALY: quality-adjusted life year.
